# Production and characterization of green and biodegradable pectin and whey protein isolate edible films

**DOI:** 10.1007/s13197-026-06791-9

**Published:** 2026-06-23

**Authors:** Enes Kavrut

**Affiliations:** Application and Research Center, Innovative Food Technologies Development, Igdir University, 76002 Igdir, Turkey

**Keywords:** Green packaging, Biodegradability, Pectin, Whey protein isolate, Edible film, Characterization

## Abstract

In this study, edible films were prepared using pectin (PEC) and whey protein isolate (WPI), and additional film formulations were developed by incorporating thyme essential oil to these polymer matrices. The films were characterized in terms of water solubility, color, thickness, opacity, contact angle, swelling ratio, water vapor permeability, antioxidant radical scavenging capacity, microstructure, green profile, and biodegradability. Pectin-based edible film groups exhibited a higher green profile score (0.84) and greater degradability, remaining stable for more than seven days. In addition, PEC-based films demonstrated superior antioxidant radical scavenging activity (7.83%), higher transparency (0.07–0.21 A.mm^−1^), and complete water solubility (100%). In contrast, WPI-based film groups were lighter in color (88.66–88.75), thicker (0.55–0.78 mm), and showed lower water vapor permeability (0–1 × 10^–5^ gm^−1^ Pa^−1^ s^−1^). Overall, the findings suggest that pectin is a more suitable polymer than WPI for the development of edible films with enhanced functional and environmentally friendly properties.

**Supplementary Information:**

The online version contains supplementary material available at https://doi.org/10.1007/s13197-026-06791-9.

## Introduction

Plastic films occupy a major share of the food packaging sector due to their strong barrier and mechanical properties, as well as their low productions costs. However, petroleum-based plastic films are highly resistant to biodegradation in natural environments, resulting in a significant environmental issue commonly referred to as “white pollution” (Haosagul et al. 2019). As fourth-generation packaging material, biopolymers have emerged as promising alternatives in food packaging because of their biodegradability, processability, eco-friendliness, and halochromic properties (Kavrut 2024). Among these materials, edible films have experienced remarkable growth over the past two decades. Food packaging generates millions of tons of landfill waste annually, creating a substantial environmental burden. Therefore, the development of biodegradable, green, and eco-friendly biopolymers has become increasingly important (Espitia et al. 2014). Recent technological advances have facilitated the production of biodegradable films derived from edible and eco-friendly sources. Consequently, considerable attention has been directed toward natural biopolymers such as pectin (PEC) and whey protein isolate (WPI). Previous studies have demostrated that pectin-based coatings can extend the shelf life of fruits, including apples and citrus fruits, by reducing moisture loss and delaying oxidative deterioration (Nastasi et al. 2022). Similarly, WPI-based films have been reported to reduce lipid oxidation by acting as effective oxygen barriers in products such as meat, milk and butter. Moreover, PEC-WPI combinations have been shown to further enhance the barrier properties of edible films (Du et al. 2022, Kavrut and Sezer 2024).

Extensive studies have been conducted in this field, leading to generation of comprehensive datasets and network analysis. In recent years, PEC- and WPI-based films have attracted considerable interest as sustainable alternatives to conventional plastic packaging. PEC is widely used in the food industry as a gelling and thickening agent. Through the formation of hydrogen bands, it creates a compact network structure that serves as an effective barrier against gases and aroma compounds. WPI is also recognized as an excellent film-forming material with superior gas barrier properties. More recently, plant-derived essential oils, such as thyme, and rosemary oils, have been incorporated into edible films as natural functional additives. These essential oils are commonly used in food packaging applications because of their antimicrobial and antioxidant activities (C. Chen et al. 2025).

Thyme (*Thymus vulgaris* L.) is a perennial plant native to the Mediterranean region and is particularly rich in polyphenolic compounds. It has been extensively investigated as a natural source of antioxidants, primarily due to the presence of thymol and carvacrol. Belonging to the Lamiaceae family, the quality and functionality of thyme are largely attributed to its essential oil composition (Ezz 2009; Gedikoğlu 2022).

In the present study, PEC and WPI, two different natural biopolymers were utilized to develop self-degradable film systems. The novelty of this study lies in the development and comparision of multifunctional packaging materials through to incorporation of thyme essential oil into different polymer matrices. Although these components have previously been investigated in separate systems, their combined application with the thyme essential oil has been limited in the literature. The films produced in were characterized in terms of their physical properties (thickness and water vapor permeability), morphological feature (surface structure), optical properties (color and transparency), and antioxidant activity (Figure S1). In addition, the biodegradability of the films was evaluated to assess their sustainability within the framework of an eco-friendly production approach. Additionally, the AGREE (Green Analysis Evaluation Approach) criterion was applied to determine the compliance of the film production methods with the principles of green chemistry.

## Materials and methods

### Materials

Thyme (*Thymus vulgaris* L.) was purchased from Baghdad Spice. The thyme, produced in 2025, was supplied in dried form and mechanically crushed (coarsely ground). Whey protein isolate (96%, Davisco Foods USA), glycerol (Sigma-Aldrich, USA), and Tween 80 were used. Apple pectin was obtained from Tito-Smart Kimya (İzmir, Turkey). All chemicals were purchased from Sigma (St. Louis, MO, USA) and Merck (Darmstadt, Germany).

### Production of edible films

The whey protein isolate film (WPIF) was produced according to the method developed by Kavrut (2024). A 5% (w/v) WPI film solution was prepared. Then, 5 mL of glycerol was added and thoroughly mixed using a magnetic stirrer. The solution was subsequently heated to 85 °C for 25 min and then cooled to room temperature. Subsequently, 25 mL of the solution was poured into Teflon pans and left to dry.

The pectin-based edible film (PEF) solution was prepared with minor modifications according to Chaichi et al. (2017). A 5% (w/v) pectin aqueous solution was stirred at 75 °C using a heatied magnetic stirrer until fully dissolved. Following dissolution, 5 mL of glycerol was added, and the mixture was stirred for 1 h. The solution was subsequently cooled to room temperature. Then, 25 mL of the solution was poured into Teflon pans and left to dry.

*Thyme essential oil-added whey protein isolate films (TWPIF):* 5 g of WPI was weighed and dissolved in 85 mL of distilled water. It was then mixed on a magnetic stirrer. The solution underwent heat treatment in a water bath at 85 °C for 25 min, followed by cooling to room temperature. An emulsion solution comprising 10 mL distilled water, 0.1 mL Tween 80, and 0.5% thyme oil was subsequently added to the WPI film solution at room temperature, and mixing was continued. Subsequently, 25 mL of the solution was poured into Teflon pans and left to dry.

*Thyme essential oil-added pectin films (TPEF):* 5 g of PEC was weighed and dissolved in 85 mL of distilled water. The solution was stirred at 75 °C using a heated magnetic stirrer. After dissolution, 5 mL of glycerol was added, and the mixture was stirred for 1 h. The solution was then cooled to room temperature. An emulsion solution (10 mL distilled water, 0.1 mL Tween 80, and 0.5% thyme oil) was added to the cooled pectin solution, and stirring was continued. Then, 25 mL of the solution was poured into Teflon pans and left to dry.

### Preparation of thyme essential oil

50 g of thyme was weighed. The crushed thyme was placed in a Clevenger-type distiller with the addition of 500 mL of distilled water. The essential thyme oil (EO) obtained after 2–3 h of distillation was stored in the refrigerator (Kavrut 2024).

### Measurement of some characterizations properties of edible films

#### Color analysis

The color (CIELAB L a* b*) of edible films was measured using a colorimeter (Konica Minolta, R-410, Osaka, Japan). The colorimeter was calibrated using a white plate surface (Kavrut 2024).

#### Thickness

The thickness of the edible films was measured using a mechanical micrometer (Astor-Türkiye) (Zolfi et al. 2014) at six random points.

#### Opacity

Edible films of uniform thickness were cut into rectangular strips measuring 1 × 4 cm. The strips were positioned flat against the inner wall of a UV–Vis spectrophotometer cuvette (Aquamate UV–Vis, China). Spectral measurements were conducted using air as the reference. Opacity was determined by measuring absorbance at 600 nm (Luz et al. 2023). The following equation was used to calculate the opacity of edible films.

$$ {\mathrm{Opacity}} \left( {{\mathrm{A}}{\mathrm{.mm}}^{{ - 1}} } \right) = {\mathrm{A}}_{{600 }} /{\mathrm{X}} $$

A_600_ is the absorbance measured at 600 nm, and X is the thickness of the edible film in millimeters (mm).

#### Contact angle

Water retention of edible films was evaluated by measuring the contact angle of water droplets on their surfaces. Films were cut into 2 × 5 cm pieces. A micropipette (Eppendorf, Germany) was used to dispense 10 μL of distilled water onto each sample from a height of 1 cm above the surface. Droplet images were captured from both sides using a digital camera (Samsung S22, Vietnam). The contact angle was calculated in AutoCAD as the angle between the tangent at the droplet's contact point and the line along the film surface (Hasheminya et al. 2018).

#### Swelling ratio

Dried samples (3 × 5 cm) were immersed in water for 30 min. After removal, the swollen samples were blotted with a paper towel and weighed (Cheng et al. 2022). The swelling test (ST) of the edible films was calculated using the following equation:

$$ {\mathrm{SR}}\left( \% \right) = \frac{{{\mathrm{W}}_{{2 }} {\text{ - W}}_{{1}} }}{{{\mathrm{W}}_{{1}} }} \times 100 $$

W_1_ is the weight of the dry edible film, W_2_ is the weight of the swollen edible film.

#### Water solubility (W_***S***_***)***

The solubility of edible films in water reflects the coating's resistance in an aqueous environment. The solubility of edible films was determined using the method described by Shakouri et al (2023) with minor modifications. First, the edible film samples (3 × 3 cm) were dried at 110 °C (Memmer UN 55plus, Germany) for 24 h. The initial solid content of the films was determined by weighing. The films were then immersed in 40 mL of water and stirred at room temperature for 6 h. The insoluble fraction was separated, dried at 110 °C, and its final weight was measured. The water solubility (W_S_) of the edible films was calculated using the following equation:

$$ {\mathrm{Water}} {\mathrm{solubility}} \left( {{\mathrm{Ws}}} \right) = \frac{{{\mathrm{W}}_{{\text{i }}} {\text{ - W}}_{{\mathrm{f}}} }}{{{\mathrm{W}}_{{\mathrm{i}}} }} \times 100 $$

W_i_ is the initial weight of the edible film sample, and W_f_ is the weight of the insoluble fraction.

#### Water vapor permeability (WVP)

The WVP of edible films was determined gravimetrically by a modified version of the standard ASTM E96-95 method. The films were cut into circles slightly larger than the diameter of the containers and sealed with rubber and paraffin. The permeability containers were filled with 3 g of CaCl_2_ to achieve a relative humidity close to 0% and weighed. After sealing, the containers were placed in a water-filled desiccator to maintain 75% relative humidity. The containers were weighed every hour for 6 h. Three replicates of each film were tested (Alvarez-Perez et al. 2022). WVP was calculated using the following equation:

$$ {\mathrm{WVP}} = \frac{{\Delta {\mathrm{m}} \times {\mathrm{L}}}}{{\Delta {\mathrm{t}} \times {\mathrm{A}} \times \Delta {\mathrm{P}}}} $$

*Δm:* Difference in water mass passing through the film over a given time (g), *A*: Film area (m^2^), *L*: Film thickness (m), *ΔP*: Water vapor partial pressure difference (Pa), *Δt:* Measurement time (seconds).

#### Antioxidant activity

The antioxidant properties of edible films were determined using the 1,1-diphenyl-2-picrylhydrazil (DPPH) assay described by Latiff and coworkers (2025), with minor modifications. 50 mg of films containing thyme oil (TWPIF, TPEF) were weighed and mixed with 10 mL of distilled water for 2 h (HZQ113 × 300, China). The mixture was then centrifuged at 4000 × g for 5 min (Beckman Coulter-ABD), and the supernatant was collected for the assessment of antioxidant activity. A 0.1 mM DPPH solution was prepared in ethanol and combined with both the film extract and a control sample. The mixture was incubated at room temperature in the dark for 30 min, after which absorbance was measured at 517 nm (Latiff et al. 2025).

The following equation was used to determine DPPH radical scavenging activity:

$$ {\text{DPPH radical scavenging }}\left( \% \right) = \left[ {1 - \left( {{\mathrm{A}}_{{\mathrm{S}}} {\text{ /A}}_{{\mathrm{C}}} } \right)} \right] \times 100 $$

A_S_ is the absorbance of the film extract at 517 nm, and A_C_ is the absorbance of the control at 517 nm.

### Microstructure

The surfaces of the edible films were examined under transmitted light using an optical microscope (Nikon SMZ 1500, Japan). Film sections measuring 2 × 2 cm were placed directly on the microscope stage and covered with glass coverslips for imaging. A 100 × objective lens was used to capture detailed microstructural features of edible films. The images were saved as 2340 × 1080-pixel crops in TIF format, and the best image from each film was selected for analysis. In light microscopy, illumination settings and microscope camera parameters were controlled and kept constant for all samples and magnification levels (Arzate-Vázquez et al. 2012).

### Evaluation of the green profile of edible films

The degree of greenness of edible films produced from plant and animal polymers was analyzed using the AGREE-Analytical GREEnness Metric Approach and Software, as proposed by Pena-Pereira et al. (2020). This system enables straightforward assessment using specialized software based on the 12 fundamental principles of greenness. It generates clear, easily interpretable color pictograms and highlights the strengths and weaknesses of the green application process. The films compared in this analysis receive a score ranging from 0.0 (lowest) to 1.0 (highest). The total score represents the average of the weight scores for the 12 fundamental principles and is displayed in the center of the graph.

### Biodegradability of edible films

The biodegradability of the edible films was evaluated using a soil burial test. A flower pot was filled with soil, and the edible films were cut into 2.5 × 2.5 cm pieces. The films were placed on the soil surface and monitored for 15 days, during which they were sprayed with water twice daily. The appearance of the films was recorded using a digital camera (Samsung S22, Vietnam), and their behavior was observed under normal environmental conditions (Riaz et al. 2020).

### Statistical analysis

Data on edible films were subjected to analysis of variance (ANOVA) using GraphPad Prism 9 and IBM SPSS Statistics 25, and Duncan’s Multiple Range Test was used to determine the mean values for sources of significant variation. Principal component analysis (PCA) was performed using SIMCA 14.1 (UMETRICS, Sweden). All edible film experiments were repeated three times (n = 3).

## Results and discussion

### Color

**Fig. 1:**
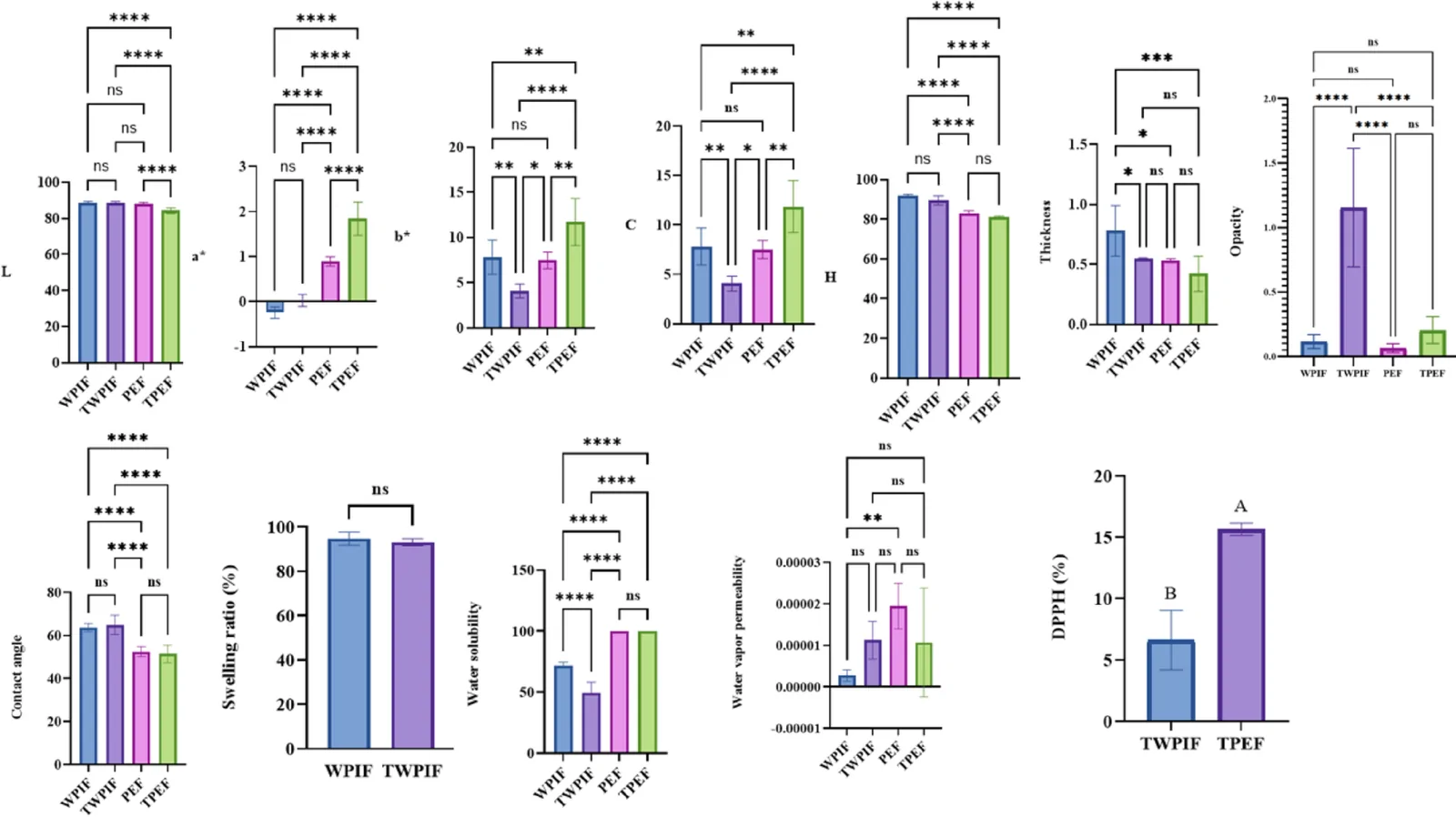
The color (L, a*, b*, C, and H), thickness, opacity, contact angle, swelling ratio (%), water solubility (%), water vapor permeability, and DPPH (%) values of edible films (WPIF, TWPIF, PEF, TPEF)

The color and appearance of edible films are critical factors influencing consumer acceptance. Ideally, edible films should be colorless and exhibit transparency comparable to synthetic packaging, while also closely matching the color of the food product (Galus and Kadzińska 2016). The transparency of emulsion-based films depends on internal factors such as the oil content in the emulsion and the size of the oil droplets (Villalobos et al. 2005). The color values of the edible films are presented in Table 1 and Fig. 1. The lowest L values were observed in the pectin groups (84.26–88.08), while the highest were found in the WPI groups (88.66–88.75). High brightness (L) values were generally detected, but these values decreased after the addition of thyme essential oil. The TPEF group recorded the lowest L value (84.26) (*P* < 0.05). This decrease in L is attributed to thyme essential oil droplets blocking light transmission through the films. Similar trends have been reported in studies conducted on different film polymers, such as soy protein, gelatin, and WPI (Galus and Kadzińska 2016). The TPEF group also exhibited the highest a* (1.84), b* (11.72), and C (11.86) values, along with the lowest H value (*P* < 0.05). These results indicate that edible films reflect light differently, which makes the product appear redder and yellower. Socaciu et al. (2020) reported that the addition of tarragon essential oil to WPI-based films reduced the L value and increased the a* value. Nikbakht et al. (2025) found that the addition of thyme essential oil to pectin and WPI films resulted in a decrease in brightness (L) and an increase in the a* and b* values.

**Table 1:** Some characterization properties (L, a*, b*, C, H, Opacity, Swelling ratio, WS, Thickness, WVP, DPPH, and Contact angle) values of edible film groups (WPIF, TWPIF, PEF, TPEF)

Groups	L	a*	b*	C (Chroma*)	H (Hue)	Opacity (A.mm^−1^)
*PEF*	88.08 ± 0.70^a^	0.89 ± 0.10^b^	7.46 ± 0.93^b^	7.51 ± 0.91^b^	83.14 ± 1.14^c^	0.07 ± 0.03^b^
*TPEF*	84.26 ± 1.44^b^	1.84 ± 0.37^a^	11.72 ± 2.61^a^	11.86 ± 2.63^a^	81.01 ± 0.65^d^	0.21 ± 0.10^b^
*WPIF*	88.75 ± 0.59^a^	-0.21 ± 0.12^d^	7.80 ± 1.88^b^	7.81 ± 1.88^b^	91.78 ± 0.76^a^	0.12 ± 0.05^b^
*TWPIF*	88.66 ± 0.68^a^	0.02 ± 0.14^c^	4.08 ± 0.77^c^	4.08 ± 0.77^c^	89.55 ± 2.33^b^	1.16 ± 0.46^a^
Groups	Swelling ratio (%)	WS (%)	Thickness (mm)	WVP (10^–5^) (gm ^−1^ Pa ^−1^ s^−1^)	DPPH (%)	Contact angle
*PEF*	*	100 ± 0.00^a^	0.53 ± 0.02^b^	2 ± 0.0^a^	*	52.50 ± 2.59^b^
*TPEF*	*	100 ± 0.00^a^	0.42 ± 0.15^b^	1 ± 0.0^ab^	7.83 ± 8.58^a^	53.00 ± 2.53^b^
*WPIF*	94.75 ± 2.95^a^	71.67 ± 2.84^b^	0.78 ± 0.21^a^	0 ± 0.0^b^	*	63.67 ± 1.97^a^
*TWPIF*	93.13 ± 1.55^a^	49.32 ± 8.69^c^	0.55 ± 0.01^b^	1 ± 0.0^ab^	3.31 ± 3.94^b^	64.83 ± 4.58^a^

^*^: Data marked was not evaluated

^**a**^^**−**^^**c**^**:** The difference between values expressed with different lowercase letters in different samples in the same column is statistically significant (P < 0.05)

### Thickness

The thickness values of the edible films are presented in Table 1 and Fig. 1. The highest values were observed in the protein-containing groups, with WPIF (0.78 mm) being the thickest film group (*P* < 0.05). No statistically significant differences were observed among the other groups (*P* > 0.05). Film thickness is an important parameter affecting water vapor permeability. Indeed, as thickness decreases, water vapor permeability increases (Table 1). The addition of essential oils reduced the thickness of the films, with the decrease observed in the TWPIF group (0.55 mm) being statistically significant (*p* < 0.05). This effect may be attributed to the structure of Tween 80 used in the thyme oil emulsion, as its large molecular size can disrupt the continuous phase of the film matrix, leading to a less dense structure (Sánchez-González et al. 2010). These findings are consistent with the results reported by Chu et al. (2019). However, some studies in the literature have reported a gradual increase in film thickness with increasing oil concentration (Li et al. 2019). Overall, previous studies indicate that the addition of oils may either increase or decrease film thickness, suggesting a variable effect depending on the film composition and formulation conditions (Chu et al. 2019).

### Opacity

Film opacity is a critical factor influencing the visual appearance of coated products. The transparency of edible films depends on multiple factors, including the internal structure formed during drying and the size of the oil droplets (Villalobos et al. 2005). The incorporation of thyme essential oil significantly increased the opacity of the edible film groups (Table 1, Fig. 1). The TWPIF group exhibited the greatest increase in opacity, reaching 1.16 mm (*P* < 0.05). This increase may be attributed to the natural color of thyme essential oil and the formation of oil droplets throughout the films. Similar trends have been reported in studies on films with various essential oil additions, including cinnamon essential oil-added pullulan-based edible films (Chu et al. 2019), bergamot essential oil-added chitosan-based films (Sánchez-González et al. 2010), and chitosan edible films containing turmeric essential oil (Li et al. 2019).

### Contact angle

The contact angle values of the edible film groups are presented in Table 1. The water contact angle of edible films is commonly used to evaluate their hydrophilicity or hydrophobicity. The difference between the animal- and plant-based film groups was statistically significant (*P* < 0.05). A low contact angle indicates that the films are hydrophilic. The pectin-containing groups (52.50–53.00) were found to be more hydrophilic (Fig. 1). In other words, pectin-containing groups are more prone to wetting than WPI films when exposed to an aqueous environment. Mendes et al. (2020) reported that addition of cocoa butter to pectin films increased surface hydrophobicity, resulting in a higher contact angle.

### Swelling ratio

The swelling ratios of the edible film groups are presented in Table 1. The swelling ratio of the pectin-containing films was not measured because these films completely dissolved in water, which is attributed to their high hydrophilicity and strong affinity for water. The WPI film groups exhibited a swelling ratio of over 90%, although this difference was not statistically significant (*P* > 0.05) (Fig. 1). The swelling and dissolution behavior of the protein films is related to the structure of WPI gels (Saikhwan et al. 2010). Low swelling values in edible films indicate that the films are less likely to undergo structural changes when used with foods containing high amounts of water. Basiak et al. (2015) reported that the swelling ratio of composite films increased from 34 to 71% with increasing WPI content. Similarly, Fematt-Flores et al. (2022) reported that the swelling ratio of edible films increased with the addition of WPI and WPC, reaching values of 75.85% and 324.30%, respectively.

### Water solubility (WS)

The water solubility of edible films reflects their degradability and resistance to water. The water solubility of the film groups is presented in Table 1. All pectin-containing films completely dissolved in water, whereas WPIF dissolved at 72% and TWPIF at 50% (Fig. 1). The water solubility of pectin-based edible films is higher than that of protein-based films, which can be attributed to their hydrophilic and porous structure. In contrast, WPI films exhibit a more stable structure due to intermolecular disulfide bonds (Silva et al. 2018).

### Water vapor permeability (WVP)

The WVP values for the films are presented in Table 1 and Fig. 1. The low water vapor permeability of emulsion-based films is attributed to the small size and homogeneous distribution of lipid globules. The water vapor permeability of edible films depends on several factors, including the nature of the barrier components, film structure (homogeneous, emulsion, multilayer, etc.), crystal type, shape, size, distribution of lipids, temperature, vapor pressure, physical state of water in contact with the films, and the drying stage of the films (Morillon et al. 2002). The water vapor permeability of the pectin-based edible films was higher than that of whey protein isolate films (*P* < 0.05). PEF is more hydrophilic, which facilitates interactions with water vapor molecules. The incorporation of thyme essential oil slightly reduced the water vapor permeability of the films, which can be explained by the homogeneous distribution of oil within the film matrix, thereby limiting water passage. Similar trends have been reported by Silva et al. (2018). Basiak et al. (2015) and Fematt-Flores et al. (2022) found that an increase in whey protein content creates a more regular protein network within the film, thereby partially impeding the passage of water molecules. This has been shown to result in a tendency towards reduced water vapor permeability. These results are consistent with those of the present study. Furlan et al. (2023) reported that the addition of thyme oil to pectin-based films made the film structure more irregular, resulting in increased water vapor permeability. Ghoshal (2022) found that thyme oil, when added to films in an appropriate ratio, made the film structure more compact, thereby limiting moisture transfer.

### Antioxidant radical scavenging assay

Table 1 presents the DPPH radical scavenging activity of the films (TPEF, TWPIF). Films containing thyme essential oil exhibited DPPH scavenging activity ranging from 3 to 8%. This antioxidant activity is attributed to the bioactive compounds present in thyme essential oil, such as thymol, p-cymene, and carvacrol (Meerasri et al. 2024). The DPPH scavenging activity of TPEF was significantly higher than that of TWPIF (*P* < 0.05) (Fig. 1). This difference may be due to variations in the matrix structure of the films and the interactions between the polymer and the essential oil. Incorporation of essential oil results in a more heterogeneous film matrix, thereby altering the film morphology and density. Consequently, the available surface area for interactions between antioxidant compounds and free radicals increases. Kumar et al. (2023), Akachat et al. (2025), and Azevedo et al. (2019) reported that chitosan-based films containing tulsi essential oil, pectin-based films containing lemon essential oil, and starch/WPI-based nanocomposite films containing rosemary essential oil respectively increased DPPH radical scavenging activity.

## Microstructure

**Fig. 2:**
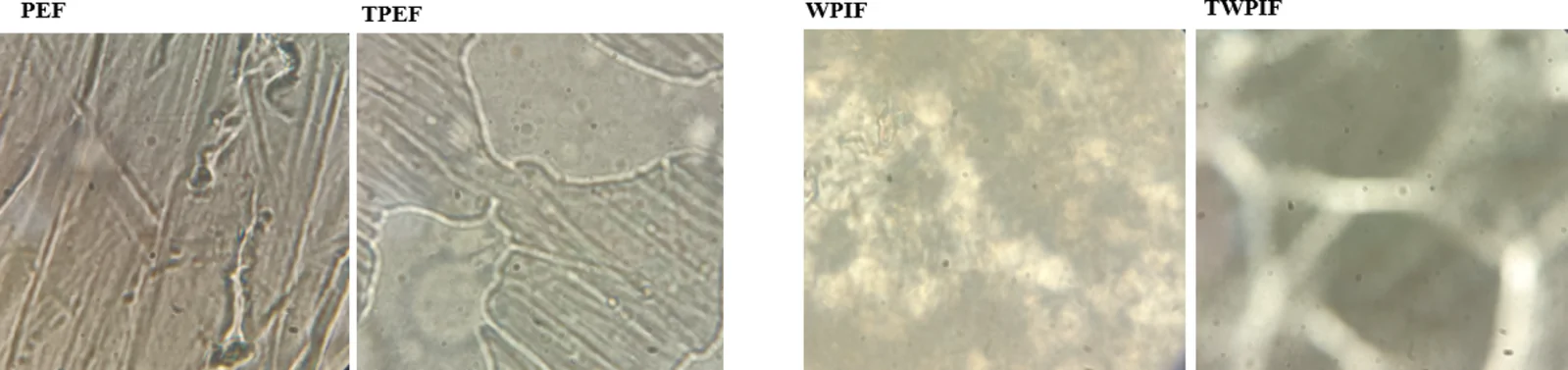
Selected micrographs of edible film groups (WPIF, TWPIF, PEF, TPEF)

The optical microscope is an effective tool for analyzing edible films, providing detailed information on their structural and surface properties (Uscanga-Sosa et al. 2025). The micrographs of surface and cross-section of the pectin-based edible films are presented in Fig. 2. The microstructure of PEF consists of long, fibrous structures. This can be explained by the pectin chains forming a regular network. It shows that the pectin chains are tightly bound through intermolecular interactions. In the microstructure of TPEF, oil droplets are seen clustered together. Whey protein isolate groups (WIPF, TWIPF) have a more compact structure. All films were observed to have a smooth structure. The more compact structure of the film matrix indicates a decrease in the water vapor permeability and solubility of composite films. Indeed, in the study, WIP groups demonstrated lower WVP and solubility in water (Table 1). The strong network structure of protein-containing films provided a more homogeneous appearance. Silva et al. (2018) reported that WPI film structure was more homogeneous than the pectin groups. The addition of thyme oil had different effects on the polymers. TPEF had larger pores and phase separation. However, the oil phase was more evenly distributed in TWPIF. This superiority of TWPIF stems from its amphiphilic property (Coşkun and Ocak 2021).

### Evaluation of the green profile of edible films

**Fig. 3:**
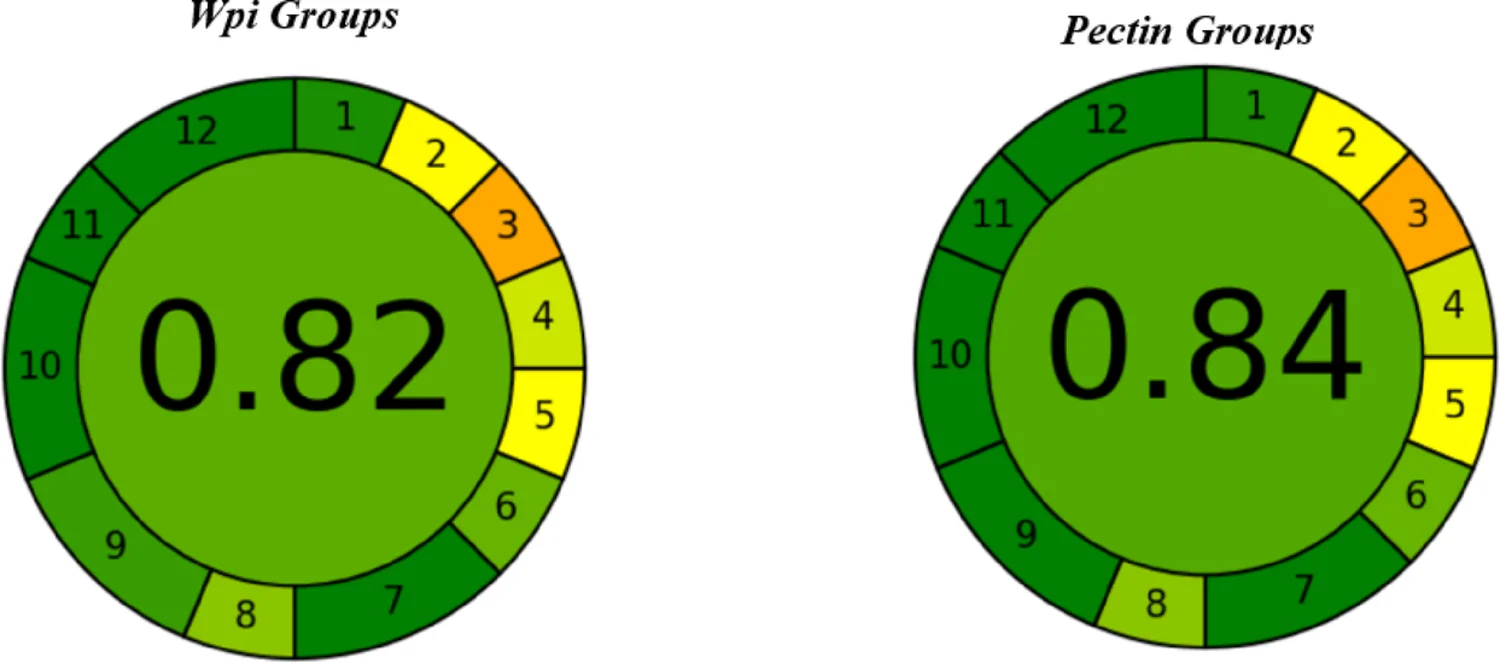
The AGREE-Analytical GREEnness Metric evaluation of edible film groups (WPIF, TWPIF, PEF, TPEF)

In this study, the AGREE Metric Approach and Software, as recommended by Pena-Pereira et al. (2020), was used to evaluate the degree of greenness of the edible films. The findings indicated that the pectin groups scored 0.84, while the WPI groups scored 0.82, demonstrating favorable green characteristics. The greenness assessment pictogram of the edible films is shown in Fig. 3. The AGREE analysis results indicate that pectin-containing films are greener than the whey protein isolate films. Pectin groups scored higher on the AGREE assessment criteria, which reflects differences in how the edible film groups met the criteria of the AGREE evaluation. The green characteristics of the edible films are influenced by multiple factors, including the polymer material used in film production, plasticizers, production process, waste generation, and energy consumption (Zhang et al. 2022).

### Biodegradability of edible films

**Fig. 4:**
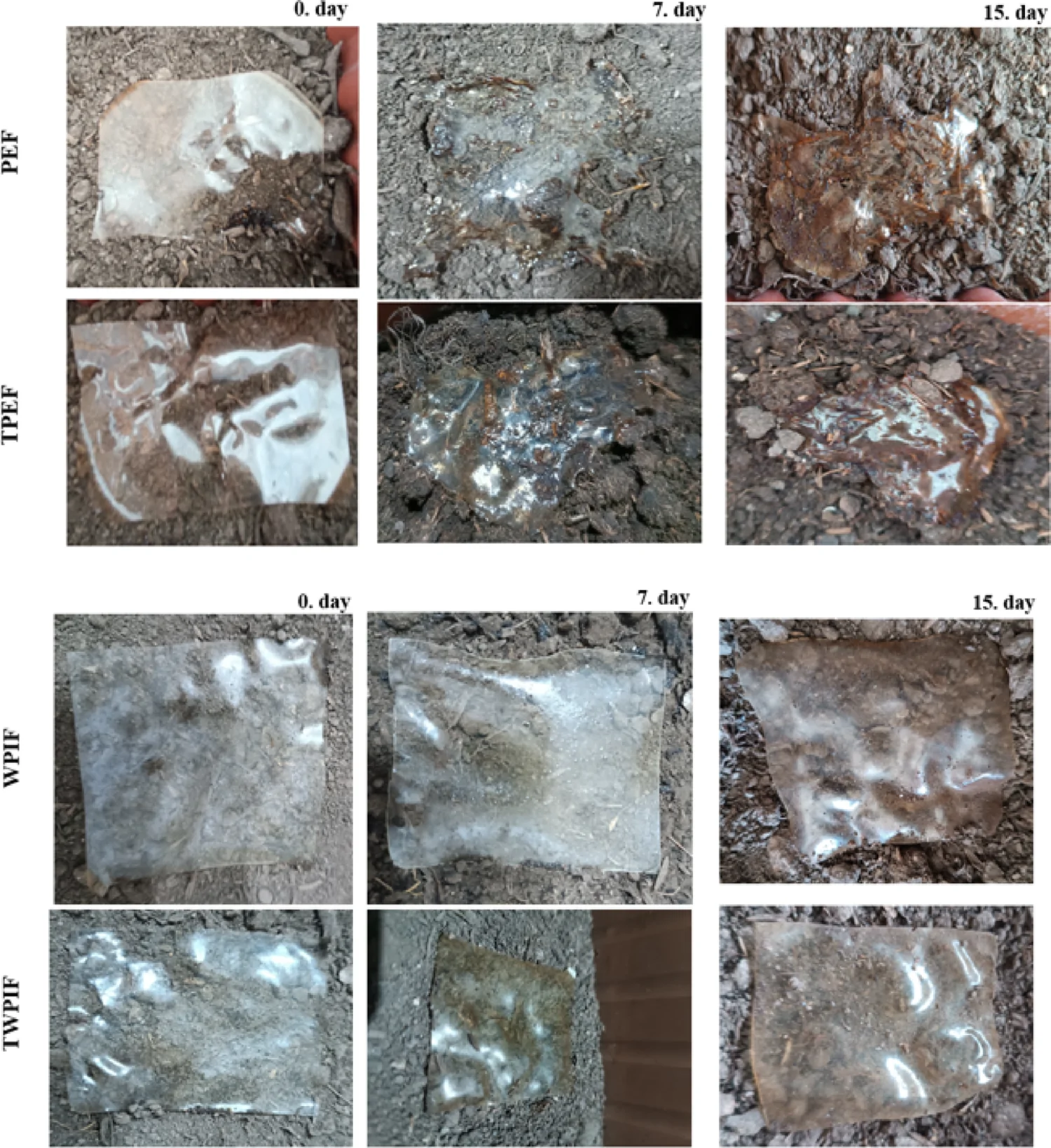
The AGREE-Analytical GREEnness Metric evaluation of edible film groups (WPIF, TWPIF, PEF, TPEF)

The images showing the biodegradation of films are presented in Fig. 4. The edible films were buried in soil, and their images were recorded on days 0, 7, and 15. Over time, rapid degradation and significant mass loss were observed in the biodegradable films. Significant differences were observed among the film groups. In particular, the pectin groups exhibited greater shrinkage and reduction from the 7^th^ day onwards, whereas this change was less pronounced in the WPI groups. By the end of day 15, pectin-based films demonstrated greater degradability compared to the WPI-based films.

Pectin-based edible films contribute to reduced environmental impact due to their enhanced biodegradability. These films undergo degradation when exposed to heat, moisture, enzymes, and environmental microorganisms. Microorganisms play a crucial role in breaking down and weakening pectin polymer chains. In the present study, edible films were formulated using protein-based (WPI groups) and carbohydrate-based (pectin groups) materials. Hydrolytic reactions promote the degradation of pectin-based films, whereas the protein content in WPI groups increases structural resistance. Covalent bonds in protein structures and the cross-linking slow down degradation. The color of the films darkened during degradation (Coltelli et al. 2015). The incorporation of thyme essential oil slightly delayed the degradability of oil-added films (TPEF, TWPIF) compared to films without oil (PEF, WIPF), which can be attributed to the effect of thyme essential oil on microbial activity. Similarly, Cerruti et al. (2011) reported that a grape-based additive affected the biodegradation rate of a starch-based polymer by reducing microbial activity.

### Principal component analysis (PCA)

**Fig. 5:**
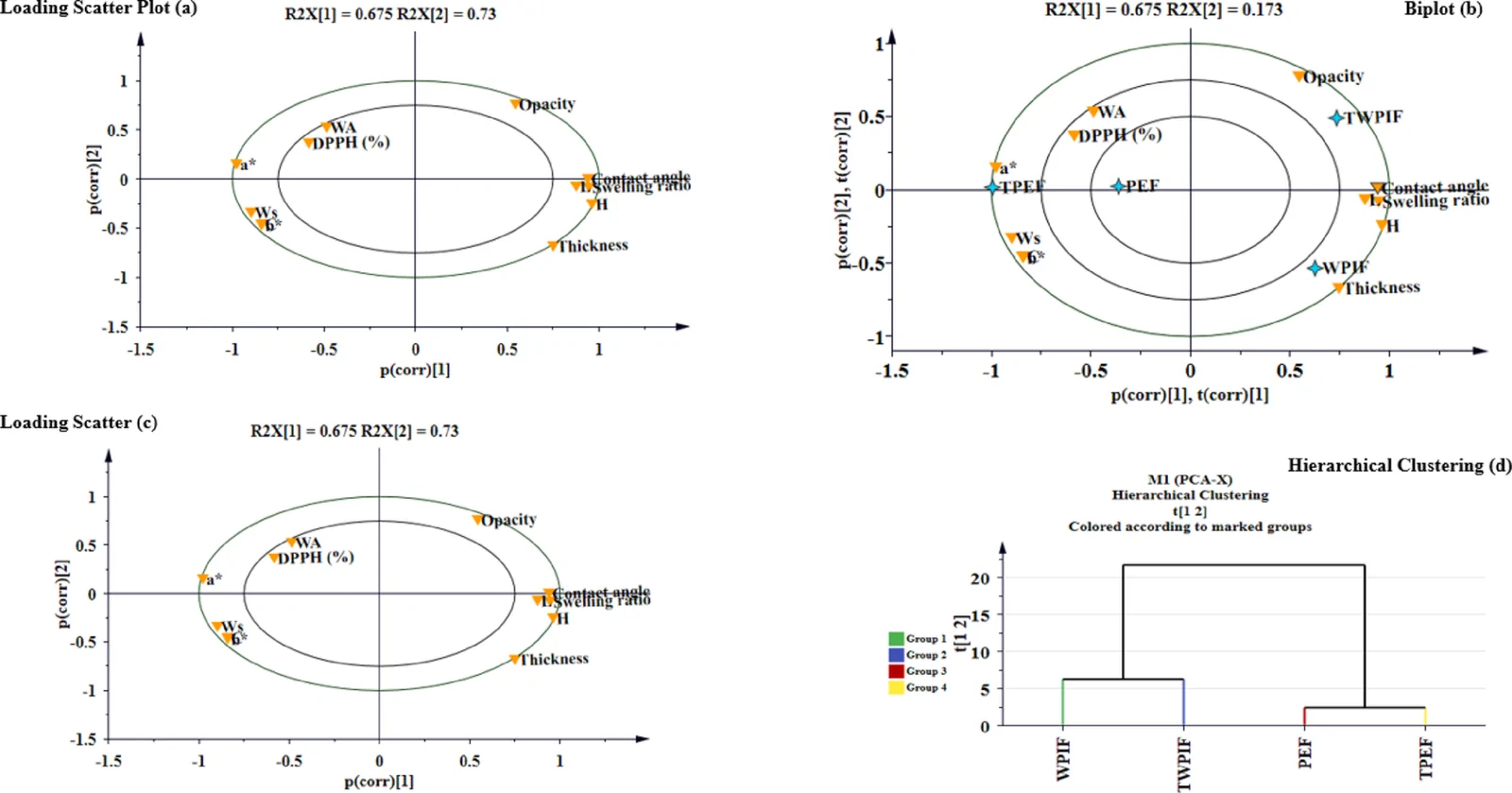
PCA graphs (Loading Scatter Plot **a**, Biplot **b**, Loading Scatter **c**, and Hierarchical Clustering **d** of edible films (WPIF, TWPIF, PEF, TPEF)

In the study, PCA was employed to reduce the dimensionality of the dataset and identify the principal components. This analysis explains a large portion of the variance in the data using a small number of components (Savaş et al. 2026). The use of PCA helped simplify the overall structure of the data and improve interpretability. PCA was applied to illustrate the differences among edible film groups by evaluating their selected characterization properties and color parameters. The score scatterplot, biplot, loading scatter plot, and hierarchical clustering of samples are shown in Fig. 5. The first two principal components (PC1 = 67.5% and PC2 = 17.3%) accounted for 84.8% of the total variance. Figure 5a shows that whilst some parameters (opacity, swelling ratio, thickness, and contact angle) exhibit similar behavior, others (DPPH (%) and color) display distinct behavior. Two distinct clusters were observed, on the left and right sides (Fig. 5c). Six analysis parameters were associated with the right cluster and six with the left cluster (Fig. 5b). The highest L, opacity, contact angle, swelling, and thickness values were observed in the WPI groups, whereas the highest a*, b* WS values were observed in the pectin groups (Fig. 5b). The clustering of the analyzed parameters is shown in Fig. 5d.

## Conclusion

In this study, green and biodegradable edible films containing pectin, whey protein isolate, and thyme essential oil were successfully produced. Films with distinct characteristics derived from plant and animal-based polymers were obtained. The characteristic properties of edible films play a crucial role in determining their physical and barrier performance. Pectin-based films exhibited features such as eco-friendly production, biodegradability, and complete water solubility, whereas protein-based films were thicker and provided a stronger barrier against water vapor transmission. These films, notable for their natural, green, and biodegradable properties, have the potential to contribute to the reduction of synthetic plastic waste in sustainable food packaging technologies.

## Supplementary Information

Below is the link to the electronic supplementary material.

Supplementary Material 1

## Data Availability

Not applicable.

## References

[CR1] Akachat B, Himed L, Salah M, D’Elia M, Rastrelli L, Barkat M (2025) Development of pectin-based films with encapsulated lemon essential oil for active food packaging: improved antioxidant activity and biodegradation. Foods 14(3):353. 10.3390/foods14030353 PMC11817409 39941946

[CR2] Alvarez-Perez OB, Ventura-Sobrevilla JM, Torres-León C, Rojas-Molina R, Rodríguez-Herrera R, Aguilar-González MA, Aguilar CN (2022) Development and characterization of whey protein films incorporated with tarbush polyphenols and candelilla wax. Food Bioscience 45:101505. 10.1016/j.fbio.2021.101505

[CR3] Arzate-Vázquez I, Chanona-Pérez JJ, Calderón-Domínguez G, Terres-Rojas E, Garibay-Febles V, Martínez-Rivas A, Gutiérrez-López GF (2012) Microstructural characterization of chitosan and alginate films by microscopy techniques and texture image analysis. Carbohydrate Polymers 87(1):289–299. 10.1016/j.carbpol.2011.07.044 34662963

[CR4] Azevedo VM, Carvalho RA, Borges SV, Claro PIC, Hasegawa FK, Yoshida MI, Marconcini JM (2019) Thermoplastic starch/whey protein isolate/rosemary essential oil nanocomposites obtained by extrusion process: antioxidant polymers. J Appl Polymer Sci 136(23):47619. 10.1002/app.47619

[CR5] Basiak E, Galus S, Lenart A (2015) Characterisation of composite edible films based on wheat starch and whey-protein isolate. Inter J Food Sci Technol 50(2):372–380. 10.1111/ijfs.12628

[CR6] Cerruti P, Santagata G, d’Ayala GG, Ambrogi V, Carfagna C, Malinconico M, Persico P (2011) Effect of a natural polyphenolic extract on the properties of a biodegradable starch-based polymer. Polymer Degradation Stability 96(5):839–846. 10.1016/j.polymdegradstab.2011.02.003

[CR7] Chaichi M, Hashemi M, Badii F, Mohammadi A (2017) Preparation and characterization of a novel bionanocomposite edible film based on pectin and crystalline nanocellulose. Carbohydrate Polymers 157:167–175. 10.1016/j.carbpol.2016.09.062 27987882

[CR8] Chakravartula SSN, Soccio M, Lotti N, Balestra F, Dalla Rosa M, Siracusa V (2019) Characterization of composite edible films based on pectin/alginate/whey protein concentrate. Materials 12(15):2454. 10.3390/ma12152454 PMC6696009 31374873

[CR9] Chen C, Ding Y, Sun Y, Li X, Sun C, Guo F, Zeng X, Gong H, Fan X (2025) Chitosan/pullulan edible coatings containing thyme essential oil nanoemulsion: preparation, characterization and application in strawberry preservation. Inter J Bio Macromolecules 309:143043. 10.1016/j.ijbiomac.2025.143043 40220827

[CR10] Cheng M, Yan X, Cui Y, Han M, Wang X, Wang J, Zhang R (2022) An eco-friendly film of pH-responsive indicators for smart packaging. J Food Eng 321:110943. 10.1016/j.jfoodeng.2022.110943

[CR11] Chu Y, Xu T, Gao C, Liu X, Zhang NI, Feng X, Liu X, Shen X, Tang X (2019) Evaluations of physicochemical and biological properties of pullulan-based films incorporated with cinnamon essential oil and Tween 80. Inter J Bio Macromolecules 122:388–394. 10.1016/j.ijbiomac.2018.10.194 30385340

[CR12] Coltelli M-B, Wild F, Bugnicourt E, Cinelli P, Lindner M, Schmid M, Weckel V, Müller K, Rodriguez P, Staebler A (2015) State of the art in the development and properties of protein-based films and coatings and their applicability to cellulose based products: an extensive review. Coatings 6(1):1. 10.3390/coatings6010001

[CR13] Coşkun AEİ, Ocak ÖÖ (2021) Foaming behavior of colloidal whey protein isolate micro-particle dispersions. Colloids Surfaces A: Physicochem Eng Aspects 609:125660. 10.1016/j.colsurfa.2020.125660

[CR15] Du Q, Zhou L, Lyu F, Liu J, Ding Y (2022) The complex of whey protein and pectin: Interactions, functional properties and applications in food colloidal systems–a review. Colloids Surfaces B: Biointerfaces 210:112253. 10.1016/j.colsurfb.2021.112253 34883341

[CR16] Espitia, P. J. P., Du, W.-X., de Jesús Avena-Bustillos, R., Soares, N. de F. F., & McHugh, T. H. (2014). Edible films from pectin: Physical-mechanical and antimicrobial properties-A review. *Food Hydrocolloids*, *35*, 287–296. 10.1016/j.foodhyd.2013.06.005

[CR17] Ezz, A. L. (2009). Plant growth strategies of *Thymus vulgaris* L. in response to population density. *Industrial crops and products*, *30*(3), 389–394. 10.1016/j.indcrop.2009.07.008.

[CR18] Fematt-Flores, G. E., Aguiló-Aguayo, I., Marcos, B., Camargo-Olivas, B. A., Sánchez-Vega, R., Soto-Caballero, M. C., ... & Rodríguez-Roque, M. J. (2022). Milk protein-based edible films: Influence on mechanical, hydrodynamic, optical and antioxidant properties. *Coatings*, *12*(2), 196. 10.3390/coatings12020196.

[CR19] Furlan GR, Silvestre WP, Baldasso C (2023) Pectin-based films with thyme essential oil: production, characterization, antimicrobial activity, and biodegradability. Polímeros 33(3):e20230029. 10.1590/0104-1428.20230053

[CR20] Galus, S., & Kadzińska, J. (2016). Moisture sensitivity, optical, mechanical and structural properties of whey protein-based edible films incorporated with rapeseed oil. *Food Technol Biotechnology*, *54*(1), 78–89. 10.17113/ftb.54.01.16.3889 PMC5105629 27904396

[CR21] Gedikoğlu A (2022) The effect of *Thymus vulgaris* and *Thymbra spicata* essential oils and/or extracts in pectin edible coating on the preservation of sliced bolognas. Meat Science 184:108697. 10.1016/j.meatsci.2021.108697 34687928

[CR22] Ghoshal G (2022) Thyme essential oil nano-emulsion/Tamarind starch/Whey protein concentrate novel edible films for tomato packaging. Food control 138:108990. 10.1016/j.foodcont.2022.108990

[CR23] Haosagul S, Boonyawanich S, Pisutpaisal N (2019) Biomethane production from co-fermentation of agricultural wastes. Inter J Hydrogen Energy 44(11):5355–5364. 10.1016/j.ijhydene.2018.09.080

[CR24] Hasheminya S-M, Mokarram RR, Ghanbarzadeh B, Hamishekar H, Kafil HS (2018) Physicochemical, mechanical, optical, microstructural and antimicrobial properties of novel kefiran-carboxymethyl cellulose biocomposite films as influenced by copper oxide nanoparticles (CuONPs). Food Packaging Shelf Life 17:196–204. 10.1016/j.fpsl.2018.07.003

[CR25] Kavrut, E., & Sezer, Ç. (2024). The antimicrobial effect of edible film packaging in instant meatballs on *Escherichia coli* O157: H7. *J the Hellenic Veterinary Med Soc*, *75*(1), 7059–7072. 10.12681/jhvms.34084.

[CR26] Kavrut, E. (2024). Production of pH-Sensitive natural decomposition Indicator with whey protein isolate-based Edible Film to monitor Fish Freshness. *Latin Am Appl Res*, *54*(2), 247–252. 10.52292/j.laar.2024.3098

[CR27] Kumar H, Ahuja A, Kadam AA, Rastogi VK, Negi YS (2023) Antioxidant film based on chitosan and tulsi essential oil for food packaging. Food Bioprocess Technol 16(2):342–355. 10.1007/s11947-022-02938-6

[CR28] Latiff NF, Sulaiman NF, Shaik MI, Mohamad NJ, Khairul WM, Daud AI, Sarbon NM (2025) Halochromic smart film: a gelatin-based pH-sensitive film embedded with anthocyanin from roselle (Hibiscus sabdariffa) extracts for potential food spoilage indicator application. J Food Sci 90(3):e70134. 10.1111/1750-3841.70134 PMC11924884 40111016

[CR29] Li Z, Lin S, An S, Liu L, Hu Y, Wan L (2019) Preparation, characterization and anti-aflatoxigenic activity of chitosan packaging films incorporated with turmeric essential oil. Inter J Biological Macromolecules 131:420–434. 10.1016/j.ijbiomac.2019.02.169 30831165

[CR30] Luz RF, Ferreira RDR, Silva CNS, Miranda BM, Piccoli RH, Silva MS, Paula LC, Leles MIG, Fernandes KF, Cruz MV (2023) Development of a halochromic, antimicrobial, and antioxidant starch-based film containing phenolic extract from jaboticaba peel. Foods 12(3):653. 10.3390/foods12030653 PMC9914361 36766181

[CR31] Meerasri J, Sukatta U, Rugthaworn P, Klinsukhon K, Khacharat L, Sakayaroj S, Chollakup R, Sothornvit R (2024) Synergistic effects of thyme and oregano essential oil combinations for enhanced functional properties of sericin/pectin film. Inter J Biological Macromolecules 263:130288. 10.1016/j.ijbiomac.2024.130288 38378108

[CR32] Mendes JF, Norcino LB, Manrich A, Pinheiro ACM, Oliveira JE, Mattoso LHC (2020) Characterization of pectin films integrated with cocoa butter by continuous casting: Physical, thermal and barrier properties. J Polymers Environ 28(11):2905–2917. 10.1007/s10924-020-01829-1

[CR33] Morillon V, Debeaufort F, Blond G, Capelle M, Voilley A (2002) Factors affecting the moisture permeability of lipid-based edible films: a review. Critical Reviews Food Sci Nutrition 42(1):67–89. 10.1080/10408690290825466 11833637

[CR34] Nastasi JR, Kontogiorgos V, Daygon VD, Fitzgerald MA (2022) Pectin-based films and coatings with plant extracts as natural preservatives: a systematic review. Trends Food Sci Technol 120:193–211. 10.1016/j.tifs.2022.01.014

[CR35] Nikbakht, H., Rajaei, A., & Movahednejad, M. H. (2025). Investigating physical and mechanical properties of edible film based on pectin and whey protein, containing thyme essential oil using Pickering nanoemulsion method. *J food sci technol (Iran)*, *22*(164), 132–156. 10.22034/FSCT.22.164.132

[CR36] Pena-Pereira F, Wojnowski W, Tobiszewski M (2020) AGREE—analytical GREEnness metric approach and software. Analytical Chem 92(14):10076–10082. 10.1021/acs.analchem.0c01887 PMC7588019 32538619

[CR37] Riaz A, Lagnika C, Luo H, Dai Z, Nie M, Hashim MM, Liu C, Song J, Li D (2020) Chitosan-based biodegradable active food packaging film containing Chinese chive (Allium tuberosum) root extract for food application. Inter J Biological Macromolecules 150:595–604. 10.1016/j.ijbiomac.2020.02.078 32057859

[CR38] Saikhwan P, Mercadé-Prieto R, Chew YMJ, Gunasekaran S, Paterson WR, Wilson DI (2010) Swelling and dissolution in cleaning of whey protein gels. Food Bioproducts Processing 88(4):375–383. 10.1016/j.fbp.2010.09.006

[CR39] Sánchez-González L, Cháfer M, Chiralt A, González-Martínez C (2010) Physical properties of edible chitosan films containing bergamot essential oil and their inhibitory action on Penicillium italicum. Carbohydrate Polymers 82(2):277–283. 10.1016/j.carbpol.2010.04.047

[CR40] Savaş A, Kavrut E, Engin T (2026) Valorization of sour cherry seeds in beef meatballs: effect on quality, lipid oxidation, texture profile, acrylamide formation and antioxidant activity. Foods 15(5):968. 10.3390/foods15050968 PMC12985260 41829241

[CR41] Shakouri M, Salami M, Lim L-T, Ekrami M, Mohammadian M, Askari G, Emam-Djomeh Z, McClements DJ (2023) Development of active and intelligent colorimetric biopolymer indicator: Anthocyanin-loaded gelatin-basil seed gum films. J Food Measurement Characterization 17(1):472–484. 10.1007/s11694-022-01640-7

[CR42] Silva KS, Fonseca TMR, Amado LR, Mauro MA (2018) Physicochemical and microstructural properties of whey protein isolate-based films with addition of pectin. Food Packaging Shelf Life 16:122–128. 10.1016/j.fpsl.2018.03.005

[CR43] Socaciu, M. I., Fogarasi, M., Semeniuc, C. A., Socaci, S. A., Rotar, M. A., Mureşan, V., ... & Vodnar, D. C. (2020). Formulation and characterization of antimicrobial edible films based on whey protein isolate and tarragon essential oil. *Polymers*, *12*(8), 1748. 10.3390/polym12081748 PMC7464654 32764387

[CR44] Uscanga-Sosa, D. P., Pérez-Gago, M. B., Contreras_Oliva, A., Salinas-Ruiz, J., Gómez-Merino, F. C., Herrera-Corredor, J. A., & Hidalgo-Contreras, J. V. (2025). Characterization Techniques for Optical, Structural, Morphological and Biodegradation Properties of Food Films. *Agro Productividad*. 10.32854/nznqqm44

[CR45] Villalobos R, Chanona J, Hernández P, Gutiérrez G, Chiralt A (2005) Gloss and transparency of hydroxypropyl methylcellulose films containing surfactants as affected by their microstructure. Food Hydrocolloids 19(1):53–61. 10.1016/j.foodhyd.2004.04.014

[CR46] Zhang Z, Wang H, Khan S, Shu Y, Liang T (2022) A green film-forming investigation of the edible film based on funoran: preparation, characterization, and the investigation of the plasticizer effects. Foods 11(19):2971. 10.3390/foods11192971 PMC9563599 36230047

[CR47] Zolfi M, Khodaiyan F, Mousavi M, Hashemi M (2014) Development and characterization of the kefiran-whey protein isolate-TiO2 nanocomposite films. Inter J Biological Macromolecules 65:340–345. 10.1016/j.ijbiomac.2014.01.010 24418333

