# Supplementary material for: Production and characterization of green and biodegradable pectin and whey protein isolate edible films
Source: J Food Sci Technol. 2026 Jun 23;63(11):2287–97. doi: 10.1007/s13197-026-06791-9 (PMC13687490; doi:10.1007/s13197-026-06791-9)
Supplement: Supplementary file 1 [file 13197_2026_6791_MOESM1_ESM.docx]

**Figure S1**. Schematic diagram of edible film characterization processes
